# Academy of Stomatology

**Published:** 1902-04

**Authors:** 

**Affiliations:** Academy of Stomatology


### Reports of Society Meetings.

### ACADEMY OF STOMATOLOGY.

A regular meeting of the Academy of Stomatology was held at the rooms of the Academy, 1731 Chestnut Street, on the evening of Tuesday, October 22, 1901, the President, Dr. S. B. Luckie, in the chair.

*Dr. James Truman.*—Dr. Kirk, during the past summer, spent some time with Dr. Michaels in Paris, examining his investigations on saliva. I understand that Dr. Kirk is ready this evening to show some of the results, and I would move that he be requested to give his experience in Paris during that period.

During a recess Dr. Kirk gave a practical demonstration of the workings of the polariscope and microscope in connection with the specimens of saliva presented representing various diseased conditions.

(For Dr. Kirk's remarks, see page 229.)

DISCUSSION.

*The President.*—You have heard Dr. Kirk's remarks on the interesting work of Dr. Michaels. The subject is now open for discussion.

*Dr. Inglis.*—I have listened with a great deal of interest to Dr. Kirk's remarks, and it is quite evident that Dr. Michaels has been doing some valuable work. The peculiar value evidently lies in the possibilities of saliva as a diagnostic medium which is readily obtainable at all times. The broad view of these possibilities of course transcend all local considerations, and if realized will make Dr. Michaels a benefactor to the world. I must confess that when I read Dr. Michaels's paper on "The Rôle of Systemic Hyperacidity in Dental Erosion" I was a trifle disappointed, for several reasons. In the first place, Dr. Michaels, if I recollect properly, failed to write of the more important theories which had been advanced. The acid sodium phosphate theory was not mentioned at all. I tried the experiment of trickling a solution of potassium sulphocyanide over a tooth, as Dr. Michaels did, and could obtain no results whatever. I would like to ask Dr. Kirk what he saw relative to the question. It is quite possible that my technic was faulty, but I was unable to get the erosions from the experiment.

I would like to ask Dr. Kirk if Dr. Michaels related the excess of salts in the blood with the excessive deposits of calculus in the mouth. Probably he did, and I would simply like Dr. Kirk, if he can, to give any information on that point. Both of these points in connection with the erosions and excessive deposits of calculus are very interesting. For example, I had a patient, who was of the tall, thin, narrow-hipped variety, very much nervously exhausted by school work, a single woman about fifty, and evidently suffering from some form of malnutrition. Her physician told her that she had gout. That woman had excessive deposits of salivary calculus on the teeth. I would cleanse them, and in two weeks she would return with nearly as much calculus as before, so that local treatment offered little encouragement. I would like Dr. Kirk to bring out these two practical points. I do not insist on my own experiment as being any proof.

*Dr. Kirk.*—I hesitate very much to attempt an answer to these interesting questions. They are very much to the point. They are inquiries that I made of Dr. Michaels myself, and I do not want to answer them, because of the very short time I was able to give to the work, and because of the immense extent of the subject and the innumerable experiments that Dr. Michaels has been making it was quite impossible for me to get any more than a very hasty general view of the subject during the two weeks I spent with him, although I was there continuously night and day. I raised the very question that Dr. Inglis has raised with Dr. Michaels, as to whether he intended to say that the sulphocyanides, either ammonium or potassium, exerted a solvent action on the tooth-structure. The impression I gained from his explanation was not that the sulphocyanide *per se* was the solvent involved, but its existence, in greater or less proportions, was an index of the nutritional state under which the erosion took place. He felt that his article had either not fully expressed his meaning, or that it was not understood. He was astonished that that view should be taken of it, but when I confronted him with the statement that that was the only reasonable deduction from his paper, that that was the view that was held, he expressed great surprise, and I believe was on the point of preparing an essay more clearly expressing his meaning. He regarded the sulphocyanide as merely an index of the condition of the saliva, not that the sulphocyanide itself did not have a solvent action. As to the rapid formation of calculary deposits, Dr. Michaels has found that under certain conditions of nutrition, particularly in the case of excessive work and in the gouty diathesis, there is a tendency to a loss from the system of a large quantity of phosphates. That goes on for a certain time until the body, or the whole economy, as he expresses it, becomes "demineralized." There is a loss of phosphoric acid. After that has reached a certain stage, the character of the excretion changes. Instead of having a large quantity of phosphates, we find oxalates present, or there may be an excess of hydrochloric acid. I do not feel warranted in speaking with a high degree of certainty or precision in the matter, for my time was wholly taken up in the study of the saliva and the technic of examination. I had hoped to do some work on cancer. Dr. Michaels claims to be able, from specimens of the saliva, blood, and urine, to make a positive diagnosis of cancer or sarcoma. There is no doubt in his mind that the cancer blood is diagnostic, and, in connection with the urine, the saliva enables him to make a positive test. That is a very important thing, and I was hoping to pursue the subject further, but my time was limited, so that I could not go deeply into it.

*Dr. James Truman.*—In compliance, Mr. President, with your request to speak, I may say I do not feel that I know anything about the subject. It is one that requires to be worked up with the expenditure of a great deal of time. I am very glad to see that Dr. Kirk has taken it up, and has been able to give us this very satisfactory talk this evening. I was very anxious to hear his account, and that is why I made the motion. We all want to know, I think, something more about saliva than we have been familiar with in the past. To me it has been an enigma. The light that Dr. Michaels has thrown upon it, so far as he has gone, has been very satisfactory. I have no criticisms to make.

*Dr. Kirk.*—Let me say that Dr. Michaels is an American, of which fact I am glad. It always stirs up my national pride when I feel that work is being done by Americans on the other side of the water. He wants his *confrères* to have a good understanding of what he is doing. He was led to study this matter from the fact of the inability of the physicians whom he consulted to cure him of a condition of malnutrition from which he suffered for years. This led him to study his saliva, and he was astonished to find these appearances under the microscope. Then he studied it systematically, read all he could on the subject, and studied the physiological chemistry of saliva and the chemistry of nutrition. He obtained saliva from cases in the hospital, and found a common factor running through certain groups of diseases. He regards his work, even though he has done a great deal of it, merely as an entering wedge to further work which he hopes to do. It gives me great pleasure to testify to his spirit in this work. It is not of the type that is exclusive. He is not trying to do this for himself, and his office and time are at the command of any one who will express an interest in it. He says,—

"Is there anybody in America who will be interested about this? If you will only tell me the name of any one who is interested in studying this work, I will put as much time as necessary into answering letters, expounding the subject or putting it in such shape that my colleagues can utilize the information."

I am myself fitting up a laboratory for the study of saliva, and I shall be very glad to get samples of saliva of any cases of interest, with or without the history, and I shall be glad to make any investigations or aid others in doing this thing. It seems to me that it is a field that we, as dentists, should promptly occupy and develop. I think it has a distinct bearing upon the questions of erosion, pyorrhœa, and the etiology of dental caries, and, more than all, has a bearing on the question of immunity from these disorders. I shall be glad if the little beginning which has been made will be the entering wedge for some very important work.

*The President.*—If there are no further remarks, incidents of practice are in order.

*Dr. Inglis.*—Supposing that incidents of practice were to invite your attention this evening, I brought down a few specimens which, unless I am wrong, establish a new classification in tooth-fusions. In the "American Text-Book of Operative Dentistry," in Dr. Thompson's chapter upon the "Macroscopic Anatomy of the Human Teeth," there is illustrated a fusion of the third and fourth molars. I have several specimens here—one occurred in my own practice—of third molars with a fourth molar attached. These teeth are united by a true fusion of the roots only. In all previous cases of fusion I believe they are described as being attached by crowns or by crown and root. In both specimens the crowns and part of each root have been fully developed, and later during the process of development the root-pulps have fused, and from that point a common root and root-canal with one foramen have been developed. In one case the fusion with the supernumerary molar has occurred by one buccal root only, the other roots having developed as usual.

I would also report a case of arsenical necrosis. I had occasion this summer to devitalize an incisor in order to crown. I drilled from the disto-labial aspect, because of the presence of a gutta-percha filling and because there was a great deal of secondary dentine in the canal. The tooth was not in its usual axis, and the root was small, so as I drilled through the dentine I missed the canal by a short distance and perforated the side. This was done by a spear-drill, so that the perforation was slight. I supposed I had entered the pulp, and I made a small arsenical application. In the course of some days the patient returned. I found a white bleb upon the surface of the gum, about midway over the mesial surface of the root. I removed the arsenic and filled the perforation with gutta-percha, then lanced the bleb, and a certain amount of watery fluid was exuded. I syringed it out with a solution of iodine, and allowed the patient to go. Suppuration ensued. The patient was then furnished a syringe and a solution of iodine for the purpose of counteracting any possible presence of arsenic and for the purpose of stimulating and sterilizing the part. The case was brought through until this month, and in time all tissue has been regenerated; the tooth is firm, and now bears a crown. It is one of the cases which shows the self-limiting action of arsenic, and in which the tissue has regenerated after destruction by arsenic. Some three years ago I reported a case in the Academy where cataphoresis had been applied after an application of arsenic and the arsenic was forced through the apical foramen. In that case the tissue was devitalized and an ischæmic necrosis supervened with subsequent regeneration of tissue.

Adjourned.

Otto E. Inglis, D.D.S.,
*Editor Academy of Stomatology.*

